# Why does Income Relate to Depressive Symptoms? Testing the Income Rank Hypothesis Longitudinally

**DOI:** 10.1007/s11205-014-0795-3

**Published:** 2014-10-28

**Authors:** Hilda Osafo Hounkpatin, Alex M. Wood, Gordon D. A. Brown, Graham Dunn

**Affiliations:** 1 Centre for Biostatistics, Institute of Population Health, University of Manchester, Oxford Road, Manchester, M13 9PL UK; 2 Behavioural Sciences Centre, Stirling Management School, University of Stirling, Stirling, FK8 1RB Scotland, UK; 3 Manchester Centre for Health Psychology, School of Psychological Sciences, University of Manchester, Manchester, M13 9PL UK; 4 Department of Psychology, University of Warwick, Coventry, CV4 7AL UK

**Keywords:** Social rank, Relative position, Depressive symptoms, Income, Constant relative risk aversion (CRRA)

## Abstract

This paper reports a test of the relative income rank hypothesis of depression, according to which it is the rank position of an individual’s income amongst a comparison group, rather than the individual’s absolute income, that will be associated with depressive symptoms. A new methodology is developed to test between psychosocial and material explanations of why income relates to well-being. This method was used to test the income rank hypothesis as applied to depressive symptoms. We used data from a cohort of 10,317 individuals living in Wisconsin who completed surveys in 1992 and 2003. The utility assumed to arise from income was represented with a constant relative risk aversion function to overcome limitations of previous work in which inadequate specification of the relationship between absolute income and well-being may have inappropriately favoured relative income specifications. We compared models in which current and future depressive symptoms were predicted from: (a) income utility alone, (b) income rank alone, (c) the transformed difference between the individual’s income and the mean income of a comparison group and (d) income utility, income rank and distance from the mean jointly. Model comparison overcomes problems involving multi-collinearity amongst the predictors. A rank-only model was consistently supported. Similar results were obtained for the association between depressive symptoms and wealth and rank of wealth in a cohort of 32,900 British individuals who completed surveys in 2002 and 2008. We conclude that it is the rank of a person’s income or wealth within a social comparison group, rather than income or wealth themselves or their deviations from the mean within a reference group, that is more strongly associated with depressive symptoms.

## Introduction

Depression is a devastating condition which disproportionately affects low income groups (Chung et al. 2004; McBarnette 1996). The condition presents at different levels, depending on the number and severity of symptoms experienced. In addition to producing direct suffering, high levels of depressive symptoms can result in low self-esteem, relationship conflict, poor health, and suicide (Block-Joy and Hudes 2010), as well as conferring a substantial financial burden on the state (Layard 2006). A number of hypotheses have been proposed to explain why low income increases risk of high levels of depressive symptoms. The *absolute income* *hypothesis* suggests that it is the actual amount a person earns that protects them from depressive symptoms through conferring an ability to purchase goods and services that promote mental health, albeit with diminishing results (Lynch et al. 2004; Preston 1975; Rodgers 1979). In contrast, various versions of the *relative income hypothesis* (Kondo et al. 2008; Wagstaff and vanDoorslaer 2000; Wilkinson 1992, 1996) suggest that in addition to having direct effects (i.e., the ability to purchase more material goods), income may relate to depressive symptoms through the social position that it confers.

Previous studies of the income-depression relationship have indicated that the indirect effect of income (measured as an individual’s income relative to that of others within a comparison group) is important for depression (Cifuentes et al. 2008; Eibner et al. 2004; Kahn et al. 2000; Messias et al. 2011; Rai et al. 2013). However, it is not clear what specification of relative income relates to depression. In this paper we focus on one particular form of the relative income hypothesis, which proposes that it is specifically the *rank* of an individual’s income within a reference group that should matter (Wagstaff and vanDoorslaer 2000; Wood et al. 2011), and test it against an alternative specification of the relative income hypothesis in order to understand the exact mechanism through which relative income determines depressive symptoms. The *income rank hypothesis* has particular relevance to understanding the link between income and depressive symptoms because it has been suggested that individuals have an evolutionary propensity to experience depressive symptoms when they are cued to see themselves as of low social rank compared to others (Gilbert 2006; Gilbert and Allan 1998; Price et al. 1994). This income rank hypothesis (Boyce et al. 2010) is distinguished from other versions of the relative income hypotheses such as the *reference income hypothesis* according to which people are concerned with how their income compares to the mean income of a reference group. The income rank hypothesis combines social psychology research on the impact of unfavourable social comparisons on well-being (Festinger 1954) with psychiatric research showing that cognitions associated with low rank are proximal causes of depressive symptoms (Taylor et al. 2011) and primate studies showing that animals are highly sensitive to rank position (Raleigh et al. 1983; Yeh et al. 1996). Specifically, subordinate animals in competition with more dominant members of the same species have lower levels of the hormone serotonin than the dominant members (Raleigh et al. 1983; Yeh et al. 1996). For many animals, and humans over the course of evolution, these hormonal differences are believed to have conferred a survival advantage through motivating such behaviours as social withdrawal, decreased appetite and sexual drive, and hypervigilance, all of which may be appropriate reactions to being of low rank within a hostile hierarchy (Sapolsky 2004). Gilbert and colleagues (Gilbert 2006; Gilbert and Allan 1998; Price et al. 1994) have noted the similarity of these reactions to symptoms of human depression, in which serotonin is also known to play a role (Coppen and Doogan 1988; Cryan and Leonard 2000). Gilbert et al. suggest that genes predisposing to depressive symptoms have been inherited from our ancestors (Price et al. 1994), for whom depression-like behaviour in response to low social rank served as an adaptive mechanism for surviving competitive social situations. For example, by withdrawing oneself and reducing appetite and sexual behaviour so as not to compete for food and potential mates, subordinate members were able to signal a ‘no-threat’ status to the more dominant members. As a result, individuals who were able to respond in this way were more likely to survive, thus passing on their genes to future generations. While these hard-wired responses previously conferred an evolutionary advantage, such reactions to low rank can have maladaptive consequences for the individual in modern societies where having a low social rank persists over a longer duration and can result in perceptions of defeat and entrapment (Taylor et al. 2011). To the extent that social comparisons result in stress, people may also experience homeostatic responses releasing various hormones including cortisol (Hill et al. 1967; Mason 1968; Taylor et al. 2011; Wood et al. 2011); such hormones have also been associated with risk of depressive symptoms (Anda et al. 1993; Jacobs 1994; Willner and Goldstein 2001) when the response is prolonged.

Although the study of the importance of income position for various forms of well-being, including depression, has a long history (for example the Whitehall study by Martikainen et al. 2003 and more recently the study by Elgar et al. 2013), there has been no previous direct test of the income rank hypothesis concerning the relationship between income and depression that additionally controls for the effect of absolute level of income. More recently a growing body of work has assessed the effect of income rank on other indicators of well-being, whilst controlling for absolute income (Boyce et al. 2010; Daly et al. in press; Wood et al. 2011). For example, Wood et al. (2011) found that individually both income and income rank within a geographic community are related to general psychopathology. However, when general psychopathology was jointly regressed on income and rank, additionally controlling for covariates, only income rank remained a predictor. This suggests that the relationship between income and general psychopathology is better explained by income rank. However it is not clear whether these results would hold for a measure of depression specifically. More importantly, whilst Wood et al.’s (2011) methodology allows comparison of the income rank and absolute income hypotheses, the method has two key limitations. Firstly, income was logarithmically transformed before being used to predict general psychopathology. Use of a logarithmic transformation is typical when income/well-being associations are examined, and reflects the assumption that subjective well-being is a negatively accelerating function of income. However, when effects of absolute income and rank of income are compared, misleading coefficients on the relative rank variable might result if well-being is not a linear function of logarithmically transformed income (because the rank variable might capture any non-linearity). This could lead to the erroneous conclusion that the association between income and well-being is due to income rank position, when in fact the results merely reflect the fact that the true relationship between absolute income and well-being is not perfectly logarithmic. Here, in order to adequately capture the non-linear relationship between income and degree of depressive symptoms, we use the more flexible utility function commonly adopted within economics—the constant relative risk aversion (CRRA) formulation—to transform absolute income. Secondly, in previous applications of the methodology used by Wood et al. (2011), the logarithmic transformation of income, income rank, and income’s distance from the mean income of a comparison group have been entered simultaneously into regressions. While this approach has the advantage of providing a direct test of the income rank hypothesis while controlling for the predictions of rival hypotheses, the approach may be problematic due to the co-linearity between measures of absolute income and relative position (Gravelle and Sutton 2009), making it difficult to separate the effects of the two and reducing confidence in the stability of the findings. Here in contrast we compare the relative fit of the income rank model with that of the income model and distance from the mean model as a means of overcoming this limitation and determining which of the hypotheses better explain the effect of income on depressive symptoms. In adopting the approach here we therefore provide a more direct and conservative test of the income rank hypothesis as well as applying it for the first time to depressive symptoms.

Finally, earlier studies have focused mainly on the effect of income on health, while there is a lack of studies on the effect of wealth. Wealth is a measure of long-term socioeconomic position whereas income is an indicator of current socioeconomic position and more likely to vary over time. It is therefore possible that the association between wealth and health is different to that between income and wealth. There is evidence that suggests wealth is a stronger predictor of health than current income (Benzeval and Judge 2001), which may be expected since many health conditions are driven by long-term risk factors (Aittomaki et al. 2010). Using a dataset which contains measures for both income and wealth, we assessed which was the stronger predictor of depressive symptoms and used this measure in our analyses.

## Methods

### Data

Two waves of data from the Wisconsin Longitudinal Study (WLS) (1992 and 2003) and the English Longitudinal Study of Ageing (ELSA) (2004 and 2008) were analysed to examine the association between income and depressive symptoms. Both populations consisted of individuals in their middle to late adulthood. These datasets are particularly suitable for our analyses as they both contain validated measures of depressive symptoms as well as a large sample size and continuous measures of individual income.

The WLS included 10,317 randomly-sampled individuals who had graduated from high schools in Wisconsin in 1957. Individuals were re-contacted and interviewed in 1992–1993 and 2003–2005. Subjects were included in our study if they responded to questions about depressive symptoms, income, and socio-demographic factors. Subjects were excluded if any information on depressive symptoms, household income, household size or any of the employed covariates was missing. The final sample consisted of 6,494 individuals at Time 1 (62.9 % of the original sample) of which 51.7 % were females, and 4,812 individuals at Time 2 (51.3 % females)—a retention rate of 74 %. Subjects who were included in our study were generally more educated than those who were not; 31 % of subjects included in our study achieved education above high school level, while 12 % of excluded subjects achieved education above high school. The mean total annual household income was $66,586.20 for subjects included in our study and $49,133.76 for those excluded at Time 1. Individuals who were included in our study at Time 1 but excluded at Time 2 did not differ by age from those who were included at both time points, although there were proportionately fewer females and subjects with health conditions (cancer, chronic liver or heart trouble, high blood pressure) included at both Time 1 and Time 2. This was partly due to the fact that some subjects who had a physical health condition died before Time 2 (*n* = 363). A logistic regression was performed to determine whether physical health status at Time 1 predicted whether or not the subject was included in Time 2 analyses; the regression showed that cancer and chronic liver problems were significant predictors of inclusion at Time 2. The WLS sample is representative of white Americans with at least complete high school education but under-representative of African-American, Asian and Hispanic populations.

ELSA is a nationally representative cohort study of individuals aged 50 years and over living in England. Participants were interviewed every 2 years from 2002 through 2008 and data were collected in questionnaire format. All participants who completed the mail questionnaires and provided data for all demographic, economic and depressive symptoms variables as well as all the employed covariates were included in our study. The final sample for our analysis at Wave 1 (2002) and 4 (2008) consisted of 11,264 and 6,425 individuals respectively. This reflects a response rate of 46.8 % at Wave 1 and an attrition rate of 57.0 % at Wave 4. At Wave 1, the mean wage for individuals who did not provide data on depressive symptoms was lower than for participants who did provide these data. There were no differences in demographics. Subjects who were included in both waves of our study (i.e., subjects who continued through to Wave 4) were generally older, more educated and had a mean net wealth four times higher than subjects who were included at Wave 1 but were excluded at Wave 4. Table 1 presents descriptive statistics for the two populations.

**Table 1:** Summary statistics of study samples

	WLS				ELSA			
	Time 1		Time 2		Time 1		Time 2	
	*N*	%	*N*	%	*N*	%	*N*	*%*
*Gender*								
Male	3,135	48.3	2,344	48.7	4,944	43.9	2,733	42.5
Female	3,359	51.7	2,468	51.3	6,320	56.1	3,692	57.5
*Year of birth*								
WLS								
1937	104	1.6	66	1.4				
1938	1,018	15.7	716	14.9				
1939	5,068	78.0	3,792	78.8				
1940	304	4.7	238	5.0				
ELSA								
1966–1975					17	0.2	9	0.1
1956–1965					229	2	130	2
1946–1955					2,992	26.6	1,919	29.9
1936–1945					3,377	30	2,105	32.8
1926–1935					2,816	25	1,606	25
1916–1925					1,567	13.9	620	9.6
1906–1915					266	2.4	36	0.6
*Highest educational achievement*								
WLS								
High school	4,480	69	3,199	66.5				
Associate degree	181	2.8	137	2.8				
First degree	1,067	16.4	823	17.1				
Masters	581	8.9	489	10.2				
MD/PhD	185	2.8	164	3.4				
ELSA								
No qualifications					4,723	41.9	2,293	35.7
Some qualification					1,501	13.3	821	12.8
‘O’ Level/nvq1/nvq2					1,821	16.2	1,170	18.2
‘A’ level/nvq3					708	6.3	443	6.9
Higher education below degree					1,238	11	816	12.7
University degree					1,273	11.3	882	13.7
*Marital status*								
Married	5,378	82.8	3,823	79.4	6,374	56.6	3,466	54
Remarried	0	0	0	0	1,232	10.9	702	10.9
Separated	41	0.6	4	0.1	135	1.2	53	0.8
Divorced	662	10.2	457	9.5	1,028	9.1	620	9.7
Widowed	138	2.1	342	7.1	1,873	16.6	1,271	19.8
Never married	275	4.2	186	3.9	622	5.5	313	4.9
*Employment status*								
Employed	5,756	88.6	2,280	47.4	3,853	34.2	1,635	25.4
Unemployed	738	11.4	2,532	52.6	7,411	65.8	4,790	74.6
*Household income ($)*	66,586.2		69,006.94					
*Total net wealth (£)*					204,205.4		232,880.2	
*Housing Tenure*								
Owner					6,087	54	4,385	68.3
Has mortgage					2,936	26.1	930	14.5
Rent					2,120	18.8	1,027	16
Live rent free					121	1.1	83	1.3
*Retirement status*								
Retired and working	508	7.8	729	15.1	0	0	0	0
Completely retired	324	5	2,353	48.9	5,437	48.3	4,038	62.9
Not retired at all	5,662	87.2	1,730	36	5,827	51.7	2,387	37.2

In WLS, household income (in dollars) was studied. In ELSA, net wealth (in British pounds) was used instead

#### Measurement of Depressive Symptoms

Both studies used the Centre for Epidemiologic Studies Depression (CES-D) measure at both waves. The CES-D is a well-validated self-reported inventory where participants rate the frequency of depressive symptoms experienced in a week. Questions used included “how many times during the past week did you feel bothered by things that don’t usually bother you?”, “how many days during the past week did you think your life had been a failure?” and “how many days during the past week did you feel you could not shake off the blues even with the help from your family and friends?”. The measure of depressive symptoms was included as a continuous rather than binary outcome to account for the deviation of each individual from the CES-D cut off point and represent the full continuum of depressive symptoms (Wood et al. 2010). The CES-D has been shown to correlate highly with clinical ratings of depressive symptoms (McDowell and Kristjansson 1996; Radloff 1977) and to have a 100 % sensitivity and 88 % specificity for identifying individuals with clinical depressive symptoms in older populations (Beekman et al. 1997). Depression scores were standardized prior to analysis to facilitate interpretation of effect sizes.

#### Income, Wealth, Income Rank, Wealth Rank and Distance from Mean Income Measures

Both respondent and total household incomes in the last 12 months were available as continuous variables. In WLS, household income rather than respondent personal income was used due to the higher correlation of household income (both untransformed and CRRA-transformed) with depressive symptoms (*r* = −0.12 and *r* = −0.11 respectively). Since ‘unequivalized’ household income (i.e. income values prior to adjusting for household size) was more strongly correlated to depressive symptoms levels than equivalized household income, unadjusted income was used in all analyses in WLS. Similar results were obtained using both methods. An income value of 1 was allocated to respondents who had negative household incomes (*n* = 6). Household income values in WLS were transformed using the CRRA utility function below:

$$u = \frac{{y^{1 - \rho } - 1}}{1 - \rho }$$

where for values of *ρ* not equal to 1, $u$ is utility, *y* is total income and *ρ* is the elasticity of marginal utility with respect to income and is assumed to be constant, and when *ρ* is 1, the function is equal to the logarithm of income. This function has been used for example by Layard et al. (2008) to examine subjective well-being as a function of income (i.e., to illustrate how the effect of income on well-being diminishes with increasing income). Layard et al. predict well-being from estimated parameters in large empirical datasets and find that the function yields the best estimates when constant *ρ* = 1.26. For our study, we derived different specifications of the CRRA function by varying the values of *ρ* used to construct the function. We then use the specification which gives the best fit for predicting depressive symptoms as our income model. Plotting scatter graphs of utility (CRRA) against household income revealed a smooth curve, suggesting no outlying subjects. Utility scores were then standardized to have zero mean and one unit standard deviation prior to analyses.

For ELSA, net total wealth rather than total household income was used as the former was more strongly correlated with and better predicted depressive symptoms: the correlation between depressive symptoms at Time 1 and transformed total household income was *r* = −0.21 while the correlation between depressive symptoms at Time 1 and transformed net wealth was *r* = −0.26. At Time 2, the correlations between depressive symptoms and total household income and net wealth were *r* = −0.14 and *r* = −0.21 respectively. Participants with a negative value (*n* = 457) for net total wealth were allocated a net value of 1, so that these subjects could be included in the analyses. Utility scores were then calculated as described above. Plotting two-way scatter graphs for utility and net wealth revealed that there were no outlying subjects.

#### Income Rank and Wealth Rank

A relative rank measure was calculated for each individual using the formula below (Brown et al. 2008; Stewart et al. 2006):

$${\text{R}}_{i} = \frac{i - 1}{n - 1}$$

where *i* is the ranked position of the individual’s income within the reference group for WLS and *i* is the ranked position of the individual’s wealth within the reference group for ELSA, *n* is the number of people in the reference group and an individual’s relative rank is a value between 0 and 1, given by the proportion of people that have a lower income in the comparison group in WLS and the proportion of people that have lower wealth in the comparison group in ELSA.

It was assumed that people generally compare themselves to people who surround them and to those with similar characteristics. Individuals were therefore ranked in terms of their position within groups of individuals of the same gender as themselves and with similar levels of education: A 6 category exploratory variable was created to compare individuals of similar education and gender (i.e., males with only high school education, females with only high school education, males with associate-level degree, females with associate level degree, etc.). Although it is difficult to determine to whom individuals compare themselves, gender and education have been central in the formation of reference groups (Singer 1981; Subramanyam et al. 2009). As there was a sufficient number of participants in each educational group (no qualifications, some qualification, General Certificate of Education (GCE): Ordinary level (O-level), General Certificate of Education (GCE): Advanced level (A-level), below degree, university degree), a 12 category variable was created for the ELSA dataset.

#### Distance from the Mean

For each time point, the difference between the best-fitting CRRA specification for income and the best-fitting CRRA specification for mean reference income was calculated. For ELSA, the best-fitting CRRA specification for wealth was used instead.

#### Covariates

Demographic and economic measures were used as covariates. Socio-demographic variables included in the WLS study were gender, age, age squared, household size, level of highest education since high school (none [high school], associate degree, bachelor degree, masters degree, doctorate or professional degree) marital status (married, separated, divorced, widowed, never married), current employment status (employed or not employed), and retirement (not retired at all, retired and working, completely retired). From ELSA, gender, age, age squared, household size, employment status (employed or self-employed), marital status (married, remarried, legally separated, divorced, widowed, never married), educational attainment (no qualifications, some qualification, GCE ‘O’ level or National Vocational Qualification (NVQ) 1 or 2, ‘A’ level or NVQ3, below degree, university degree or NVQ 4 or NVQ 5), tenure (owner, paying mortgage, renting, living rent free), retirement (not retired, fully retired, semi-retired) were used. In WLS, a variable for the degree of negative income reported (i.e. a value of 0 for subjects with income of $0 or above and reported negative income as a positive value, ranging from $0 to $14400 for those with negative incomes) was constructed and included in the analyses.

### Statistical Analyses

Statistical analysis was performed to investigate which of income rank (as a measure of social position), transformed absolute income, or transformed deviation of absolute income from the mean income within the reference group best predicted depressive symptoms in 52–56 year olds and then depressive symptoms 10 years later in WLS. In ELSA we investigated the effect of wealth rank, transformed absolute wealth and transformed deviation of absolute wealth from mean wealth within the reference group. We first obtain the best specification for income or wealth as a predictor of depressive symptoms: Least squares regression was performed to fit models containing the effect of income or wealth and all socio-demographic covariates using different estimates of *ρ* to obtain the CRRA function that best fit the data (as determined by the log-likelihood or, equivalently, the Bayesian Information Criterion, BIC—see below).

Depressive symptoms at Time 1 was regressed on transformed income or wealth at Time 1 and Time 1 covariates. Depressive symptoms at Time 2 was then regressed on Time 1 levels of depressive symptoms and Time 1 transformed income or wealth and Time 2 covariates. The regression was then repeated to include income rank or wealth rank and demographics (without income/wealth) and then transformed deviation from the mean income or mean wealth and demographics (without income/wealth and income rank/wealth rank). For both time points, our variable for depressive symptoms was jointly regressed on transformed income or wealth and income rank or wealth rank (and demographics). Finally, our variable for depressive symptoms was simultaneously regressed on transformed income or wealth, income rank or wealth rank and transformed deviation from mean.

Goodness of fit tests were used to determine the best model explaining the income- depressive symptoms relationship in WLS and the wealth-depressive symptoms relationship in ELSA. The Bayesian Information Criterion (BIC), also known as Schwarz Information Criterion, was used to choose the best-fitting model. The BIC is a large-sample asymptotic estimator which uses the log-likelihood adjusted for the number of observations and regressors (Gravelle and Sutton 2009; Raftery 1996) to approximate the Bayesian probability of a model. The model with the lowest BIC has the highest Bayesian posterior probability and is taken as the preferred model, according to the available data. This criterion is widely used for model selection purposes, with a BIC decrease of 2 or more units indicating some evidence for the model and a decrease of 6 or more indicating strong evidence (Gravelle and Sutton 2009; Raftery 1996). It should be noted that the BIC values here do not allow for the fact that *ρ* is being estimated; the BIC estimates for the income and wealth models would be higher if this was adjusted for. The R-squared value was additionally used to examine the amount of variation captured by the model. Results from the CRRA model using the optimum value of *ρ*, as well as the model using the logarithm of income or wealth (if different to the optimum) are presented here (Table 2). The BIC, R-squared, Akaike Information Criterion (AIC, an alternative goodness of fit measure) values are also presented in the results.

**Table 2:** Comparison of test statistics for models of depressive symptoms

Predictor	f(predictor)			Rank			f(predictor) + Rank		
	BIC	AIC	R squared	BIC	AIC	R squared	BIC	AIC	R squared
*WLS*									
Time 1									
Linear : Log (income)	18,308.05	18,186.04	0.03	18,268.93	18,146.91	0.04	18,257.84	18,129.04	0.04
Standardized CRRA (ρ = 0.20)	18,277.21	18,155.20	0.04	18,268.93	18,146.91	0.04	18,277.69	18,148.90	0.04
CRRA-transformed distance from the mean	18,270.47	18,148.46	0.04	18,268.93	18,146.91	0.04	18,275.80	18,147.01	0.04
Time 2 on Time1									
Linear : Log (income)	11,736.65	11,613.56	0.29	11,726.50	11,603.40	0.29	11,729.01	11599.43	0.29
Standardized CRRA (ρ = 0.40)	11,731.77	11,608.67	0.29	11,726.50	11,603.40	0.29	11,731.62	11,602.04	0.29
CRRA-transformed distance from the mean	11,733.16	11,610.06	0.29	11,726.50	11,603.40	0.29	11,730.57	11,600.99	0.29
*ELSA*									
Time 1									
Linear : Log (net wealth)	30,836.18	30,674.94	0.12	30,795.79	30,634.55	0.12	30,800.65	30,632.08	0.12
Standardized CRRA (ρ = 0.80)	30,807.11	30,645.86	0.12	30,795.79	30,634.55	0.12	30,803.30	30,634.72	0.12
CRRA-transformed distance from the mean	30,807.11	30,645.86	0.12	30,795.79	30,634.55	0.12	30,803.30	30,634.72	0.12
Time 2 on Time 1									
Linear : Log (net wealth)	16,402.10	16,246.44	0.24	16,395.12	16,239.45	0.24	16,401.43	16,239.00	0.24
Standardized CRRA (ρ = 0.60)	16,397.38	16,241.72	0.24	16,395.12	16,239.45	0.24	16,403.86	16,241.42	0.24
CRRA-transformed distance from the mean	16,396.69	16,241.03	0.24	16,395.12	16,239.45	0.24	16,403.87	16,241.44	0.24

For each time wave, two specifications are presented to model the absolute income hypothesis [f (income)]—the logarithm of income and CRRA specification using the estimate of *ρ* that gives the lowest BIC. Each of these models and the CRRA-transformed deviation from the mean model are then compared to the income rank model to assess the best fitting model for each time point. For ELSA results using net wealth are presented

Bootstrapping was then performed to determine the statistical significance of the differences in the goodness of fit measures for two non-nested models with identical degrees of freedom. This process re-samples the distribution without computational error (Jeong 2006) producing consistent results and allowing us to confirm a model truly has higher explanatory power than an alternative model (Davidson and MacKinnon 1997; Jeong 2006). The BIC values can be informally compared for models containing either the CRRA function of income or income rank but cannot be compared using a conventional significance testing since they are produced by non-nested models. Sampling variability in the variation of the difference was therefore examined using the bootstrap (Efron and Tibshirani 1993). One thousand bootstrap samples (i.e. samples with replacement) of the same size of the original data set were obtained, and for each sample the two competing models were fitted and the BIC statistics extracted. The difference between these two BIC values was computed (that for income/wealth minus the one after fitting the effects of income rank/wealth rank) and the distribution of the differences across bootstrap samples examined. If the rank model is preferred, the true difference will be positive and we therefore report the proportion of the differences with a value greater than zero (the complement of this—the probability of the difference being equal to or less than zero–provides a one sided *p* value on which to evaluate statistical significance).

## Results

### Rank Groups and Income

**Fig. 1:**
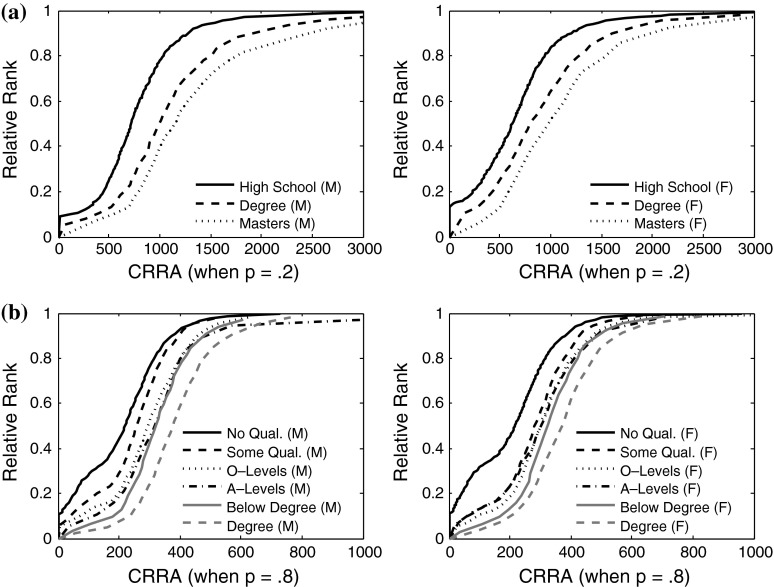
Plot of rank against constant relative risk aversion (CRRA) for the reference groups in **a** WLS and **b** ELSA. CRRA can be seen to be clearly distinguishable from rank, indicating that two individuals with the same income measure may have different ranks within their reference group. Largest vertical differences are observed at the middle of the distribution, where differences are of particular interest

In order to determine whether our value for rank was sufficiently different to the transformed measure of absolute income, scatter graphs were plotted to observe the variation in rank for the same income value and vice versa. Figure 1 shows that the range in rank for a given income level at Time 1 was considerable, with the largest range at about $40,000. Income values for a rank of 0.2, 0.4, 0.6, 0.8 and 1 ranged from approximately $16,000–$35,000, $25,000–$49,000, $34,000–$64,000, $48,000–$95000, and $145,000–$300,000 respectively at Time 1 in WLS. The plots show that transformed income and income rank are sufficiently non-linearly related to be used as different indicators. Additionally, the gaps occur mostly around the middle to bottom of the distribution, where differences between income rank and income is particularly of interest. Similar results are seen for wealth in ELSA.

### Comparison of Models

Table 2 presents the test statistics of the income model using the CRRA specification with the best-fitting value of *ρ*, the income model using the logarithm of income and the income rank model for each time point in WLS. Test statistics for the analyses using the wealth measure in ELSA are also presented in Table 2. For each time point the function with the lowest BIC for regression of depressive symptoms on income (including mentioned covariates) was selected as the best fitting model. In Table 2 we see that for WLS at Time 1, the function for income which gave the best fit was the CRRA model when *ρ* = 0.20 (*BIC*: 18277.21) rather than the model using the logarithmic function (*BIC*: 18308.05). Even when compared to the model with the best fitting function for income, a model containing rank without income had a lower BIC (*BIC*: 18268.93) and therefore overall best fit. This rank model also provided a better fit than the CRRA-transformed deviation of income from the mean income of the reference group (*BIC*: 18270.47). Table 2 shows that similar results were obtained in ELSA; In ELSA, the optimum model for regressing wealth on current depressive symptoms (Time 1) was the CRRA model when *ρ* = 0.80 (*BIC*: 30807.11). Once again, even when compared to both this specification and the CRRA-transformed deviation from the mean (*BIC*: 30807.11), the model with the lowest BIC was the rank model (*BIC*: 30795.79), supporting the wealth rank hypothesis. The rank model produced a BIC value that was 11.32 less than the BIC of the wealth model, which (under some assumptions) corresponds to a Bayes factor of 287. In other words, the odds in favour of the rank model given the data are 287. Thus, the goodness of fit statistics provide strong evidence that rank is a better predictor of current depressive symptoms. Table 3 shows that the goodness of fit test results were confirmed with the regression analyses, which showed that rank was consistently a significant predictor of current depressive symptoms for both datasets. In WLS, a one standard deviation increase in household income was associated with a 0.08 standard deviation decrease in risk of depressive symptoms, while moving from top to bottom rank (within a reference group of people of the same education and gender) reduced risk of depressive symptoms by 0.29 standard deviations and a change in CRRA-transformed distance from the mean was associated with a 0.08 standard deviation decrease in depressive symptoms. Jointly regressing the CRRA function and income rank on depressive symptoms showed that the CRRA function was no longer significant while income rank remained significant (*p* = 0.004). However, this model had a higher BIC than the models including the CRRA function and income rank alone and did not explain any more variation in depressive symptoms. This is unsurprising as BIC penalises for additional regressors. Simultaneously regressing depressive symptoms on CRRA, CRRA-transformed deviation from the mean and rank also produced a significant coefficient on the rank variable (*p* = 0.010) and CRRA-transformed distance from the mean (*p* < 0.001), although this regression indicated multi-collinearity in the predictors since the CRRA function became significant in the opposite direction. Similarly, in ELSA, jointly regressing the CRRA-transformed wealth and rank on depressive symptoms resulted in CRRA-transformed wealth losing significance, while moving from bottom to top rank was associated with a 0.32 (*p* < 0.001) standard deviation decrease in depressive symptoms. Regressing depressive symptoms on CRRA-transformed wealth, CRRA-transformed deviation from the mean and rank jointly suggested multi-collinearity as CRRA-transformed deviation from the mean became significant in the opposite direction.

**Table 3:** Estimation of adjusted coefficients using best statistical model for predicting current and future depressive symptoms in (a) WLS and (b) ELSA

Predictor variables	Depressive symptoms				
	1	2	3	4	5
*Time 1*					
(a) Standardized CRRA	−0.08			−0.00	0.28
	(0.014;0.000)			(0.029;0.898)	(0.083;0.001)
Rank		−0.29		−0.28	−0.25
		(0.045;0.000)		(0.096;0.004)	(0.096;0.010)
CRRA-transformed distance from the mean			−0.08		−0.29
			(0.013;0.000)		(0.080;0.000)
(b) Standardized CRRA	−0.16			−0.05	−0.14
	(0.016;0.00)			(0.035;0.177)	(0.033;0.000)
Rank		−0.43		−0.32	−0.32
		(0.040;0.000)		(0.089;0.000)	(0.089;0.000)
CRRA-transformed distance from the mean			−0.16		0.03
			(0.016;0.000)		(0.211;0.000)
*Time 2 on Time 1*					
(a) Depressive symptoms (T1)	0.50	0.50	0.50	0.50	0.50
	(0.012;0.000)	(0.012;0.000)	(0.012;0.000)	(0.012;0.000)	(0.012;0.000)
Standardized CRRA (T1)	−0.03			0.07	−0.09
	(0.014;0.025)			(0.036;0.067)	(0.103;0.400)
Rank (T1)		−0.14		−0.33	−0.36
		(0.044;0.001)		(0.114;0.003)	(0.115;0.002)
CRRA-transformed distance from the mean			−0.03		0.16
			(0.014;0.040)		(0.101;0.116)
(b) Depressive symptoms (T1)	0.39	0.39	0.39	0.39	0.39
	(0.012;0.000)	(0.012;0.000)	(0.012;0.000)	(0.012;0.000)	(0.012;0.000)
Standardized CCRA (T1)	−0.03			0.00	0.23
	(0.015;0.024)			(0.029;0.866)	(0.151;0.132)
Rank (T1)		−0.13		−0.14	−0.13
		(0.048;0.007)		(0.096;0.131)	(0.096;0.161)
CRRA-transformed distance from the mean			−0.04		−0.23
			(0.015;0.016)		(0.150;0.133)

(1) Model containing income/wealth + mentioned covariates (2) Model containing rank + mentioned covariates (3) Model containing CRRA-transformed distance from mean + mentioned covariates (4) Model containing income/wealth + rank + mentioned covariates (5) Model containing income/wealth + rank + CRRA-transformed distance from mean + mentioned covariates. For each predictor variable, the top row provides the estimate of the regression coefficient, the row beneath shows the corresponding (standard error;* p* value)

In Table 2 we show that the income model which best predicted future depressive symptoms from Time 1 income was the CRRA model when *ρ* = 0.40 for WLS (*BIC*: 11731.77). This model had reduced fit when compared to the model containing income rank alone (*BIC*: 11726.50). Consistently in ELSA, the optimum wealth model (the CRRA specification when *ρ* = 0.60) proved to fit slightly less well than the rank model (*BIC*: 16397.38 and 16395.12 respectively). For both datasets, the rank model consistently better predicted future depressive symptoms than did CRRA-transformed distance from the mean. As before, the results of the regression analyses in Table 3 confirmed the results. For WLS, joint regression of the CRRA function and income rank resulted in the CRRA function losing significance and rank remaining significant. Similarly, jointly regressing future depressive symptoms on CRRA, CRRA-transformed deviation from the mean and rank resulted in only rank remaining a significant predictor. However, in ELSA joint regression resulted in the CRRA function, CRRA-transformed deviation from the mean and rank losing significance, indicating multi-collinearity. We counteract this with a bootstrapping test to assess the probability that the rank model is a better fit than the income model (i.e. a BIC difference of > 0). Results showed that the proportion of the bootstrapped BIC differences greater than 0 for current depressive symptoms was 0.95 and 0.88 for WLS and ELSA respectively, providing some evidence that the rank model is generally better than the income or wealth model. For predicting future depressive symptoms, the proportion of the bootstrapped BIC differences greater than 0 was 1.00 and 0.82 for WLS and ELSA respectively.

Contingency analyses were performed to check the robustness of results. Regression was repeated excluding subjects with negative income values. Results obtained were similar, although effect sizes of rank were slightly larger in ELSA. Furthermore, the effect of rank within the overall population was also examined. Similar results were found at all time periods as when stratifying rank by gender and education. Similarly, consistent results were observed for the regression analyses using total personal income in both datasets and total household income in ELSA (though household income was not a significant predictor of future depressive symptoms in ELSA). Regressions were also repeated using depressive symptoms as a dichotomised variable and with income rank by education and yielded consistent results. Additionally, the regressions at both time points were repeated to include physical health variables (at Time 1), which did not attenuate the results. Probability weights were also created and used in the regression to predict future depressive symptoms, in order to account for the fact that physical health was a predictor of inclusion at Time 2 analyses. The latter produced similar results. Finally, inverse probability weighting was also used to handle missing data for the variables of interest at Time 1 and Time 2; we first created a variable to indicate whether data for depressive symptoms at Time 1 were missing. Regressing this variable on CRRA, rank, CRRA-transformed distance from the mean and demographic variables indicated that rank, distance from the mean, household size, gender, education, marital and retirement status predicted whether the respondent provided data on their depressive symptoms. The variable indicating whether data on depressive symptoms were missing was then regressed on these significant predictors and the inverse probability of this regression was stored and used as probability weights in our complete case regression analysis. The results using these weights were similar to those obtained using complete case analysis.

## Discussion

The results provide the first direct evidence that the relationship between income and depressive symptoms is best explained by an individual’s income rank position within a reference group. We provide a strict test of this income rank hypothesis by accounting for the direct effect of various functions of income. We show that the CRRA function represents the effect of income on depressive symptoms better than the logarithmic function, highlighting the need to fully control for the exact form of the relationship between income and well-being when comparing absolute income against relative income specifications. Consistent with the results from the study by Gravelle and Sutton (2009), we find that addition of the rank variable to the model containing the CRRA specification does not improve the fit of the model and in the case of ELSA, results in both wealth and rank losing significance. This result is consistent with Gravelle and Sutton’s conclusion that the high correlation between different indicators of socioeconomic status will present a problem in many datasets. We therefore suggest that the best way to assess whether rank has an effect on depressive symptoms above and beyond the direct effect of income or wealth is to compare the fit of two theoretical models. Here we show that the model with the lowest BIC for all time points in both WLS and ELSA was the rank model. This was confirmed with bootstrapping and sensitivity analyses. We therefore conclude that the income rank model (and wealth rank model in ELSA) is statistically and theoretically a better model for risk of depressive symptoms, providing evidence to support Marmot’s argument of the role of psychosocial factors on individual health (Marmot and Wilkinson 2001).

The study has a number of advantages—the use of a pooled dataset, reported actual values of income rather than income categories, and use of a reliable measure of depressive symptoms. Although a self-report measure of depressive symptoms is used here, the CES-D has been shown to have high sensitivity and specificity, allowing those at risk of clinical depressive symptoms to be identified. Furthermore the analysis was conducted on two datasets to see if the results were consistent in different populations and therefore generalizable to mid-life populations. Similarly, we use two different measures of utility (wages and net wealth) and find consistent results. An obvious limitation which must be considered is that it is difficult to know exactly to whom people compare themselves (Pham-Kanter 2009). More suitable reference groups could be one’s social circle or work colleagues, rather than people of similar education or gender. More accurate results could be obtained using questionnaires that inquire about reference groups or defining exact metrics along which the participants make social comparisons and including members of these social comparison groups as participants (Pham-Kanter 2009).

Through this study we show that the income-depressive symptoms relationship is likely due to psychosocial factors rather than material factors. Although we find that income rank is a better predictor of depressive symptoms than income, it must be noted that rank still explains a relatively small percentage of variability in depressive symptoms (4 and 12 % in WLS and ELSA respectively). Income, however specified, only explains a small amount of well-being relative to psychological characteristics (Boyce and Wood 2010; Boyce et al. 2013; Osafo Hounkpatin et al. 2014). Furthermore, the current research suggests an explanation of the Easterlin paradox (Easterlin 1973; Easterlin et al. 2010) where whilst income within a country is related to well-being at a point in time, increases in national income do not relate to aggregate increases in well-being. If the income/well-being relationship is better represented by rank position, at a given time point those of higher rank will have higher well-being, but increasing population income will have no impact on national well-being as there will still by definition be the same proportion of people at high and low rank. Taken together, whilst the current results provide an explanation as to why the well-observed income-depressive symptoms relationships exists, and what it represents, greater improvements in well-being may be achieved through focusing on improving social good rather than economic success.
